# Four decades of heart–lung transplantation: Milestones and outcomes in advanced cardiorespiratory failure

**DOI:** 10.1002/ehf2.15411

**Published:** 2025-08-27

**Authors:** L. Lily Rosenthal, Anna Maria Mühlbauer, Carola Grinninger, Nikolaus A. Haas, Heinrich Netz, Robert Dalla Pozza, Jürgen Hörer, Sebastian Michel, R. Schramm, D. Graetz, Christian Hagl

**Affiliations:** ^1^ Division of Paediatric and Congenital Heart Surgery LMU University Hospital Munich Munich Germany; ^2^ Department of Cardiac Surgery LMU University Hospital Munich Munich Germany; ^3^ Department of Internal Medicine Clinic Bogen Bogen Germany; ^4^ Division of Paediatric Cardiology and Intensive Care LMU University Hospital Munich Munich Germany; ^5^ Department of Cardiovascular Diseases Munich Heart Alliance (MHA) – German Centre for Cardiovascular Research (DZHK), LMU University Hospital Munich Munich Germany; ^6^ Clinic for Thoracic and Cardiovascular Surgery Heart and Diabetes Centre North Rhine‐Westphalia (HDZ NRW) Bad Oeynhausen Germany; ^7^ Clinic Gauting, Department of Intensive Care Sleep and Respiratory Medicine Gauting Germany

**Keywords:** Bronchiolitis obliterans syndrome, Congenital heart disease, End‐stage cardiopulmonary failure, Graft preservation, Heart–lung transplantation, Immunosuppression, Pulmonary hypertension

## Abstract

**Aims:**

Heart–lung transplantation (HLTx) remains a life‐saving intervention for patients with end‐stage cardiopulmonary failure. We retrospectively analysed long‐term HLTx outcomes at our centre to assess survival trends and evaluate the impact of evolving immunosuppressive, surgical and perioperative strategies.

**Methods and results:**

This single‐centre retrospective cohort study included 80 patients who underwent HLTx between 1983–1995 (Era 1) and 1996–2010 (Era 2), with follow‐up through June 2024. All patients had severe cardiorespiratory failure. The primary endpoint was all‐cause mortality. Secondary endpoints included early and late post‐transplant outcomes. Overall survival at 1, 5 and 10 years post‐transplant was 60 ± 6%, 46 ± 6% and 35 ± 6%, respectively. Survival improved significantly between Era 1 (46 ± 10%, 18 ± 9% and 9 ± 6%) and Era 2 (66 ± 7%, 5 ± 7% and 45 ± 7%) (*P* < 0.001), correlating with advancements in immunosuppression, organ preservation and perioperative care. Univariable risk factors for increased mortality included Euro Collins versus Perfadex lung preservation (*P* < 0.001), University of Wisconsin (UW2) versus Histidine‐Tryptophan‐Ketoglutarate (HTK) solution cardioplegia (*P* < 0.001), and Epstein–Barr virus infection (*P* = 0.036). Heart failure: OR 4.557 (95% CI: 1.057–19.648, *P* = 0.042) and gastrointestinal bleeding: OR 2.739 (95% CI: 1.310–5.726, *P* = 0.016) were identified as risks for mortality. These factors remained significant in multivariable analysis.

**Conclusions:**

HLTx outcomes at our centre are consistent with international benchmarks. Survival has improved in Era 2, likely due to individualised immunosuppressive regimens, novel organ preservation techniques and enhanced surveillance. These results support ongoing optimisation of multidisciplinary care for complex cardiopulmonary failure.

## Introduction

Over the past four decades, our centre's experience with heart‐lung transplantation (HLTx), including advances in immunosuppression and graft preservation, reflects the progress made globally. The first HLTx was performed in 1967.^1^ Denton Cooley's 1968 HLTx patient survived approximately 14 h, with early post‐operative survival rarely exceeding 30 days. The introduction of ciclosporin A in the late 1970s significantly improved outcomes. Reitz and colleagues at Stanford transplanted a patient with end‐stage pulmonary arterial hypertension (PAH) in 1981,^2^ and our centre conducted its first HLTx in 1983.^3^ Januszewska et al. reported outcomes of HLTx in pulmonary atresia at our centre.^4^

PAH, arising from congenital defects or idiopathic causes, remains the principal indication for HLTx. While HLTx for cystic fibrosis has declined, cases of idiopathic interstitial pneumonia have increased,^5^ partly due to the success of bilateral lung transplantation (BLTx) in idiopathic PAH.^6^, ^7^ Surgical advances now permit single lung transplantation for some patients with congenital heart defects and pulmonary hypertension. PAH is defined as a mean pulmonary arterial pressure ≥25 mmHg at rest, classified according to capillary wedge pressure and cardiac output.^8^ Hoeper et al. demonstrated the efficacy of sotatercept in idiopathic PAH.^9^ Li et al. demonstrated in their study that a combined echocardiographic model, integrating right ventricular (RV) global wasted work, RV longitudinal strain indexed to pulmonary artery systolic pressure (PASP), and tricuspid annular plane systolic excursion (TAPSE), improves risk stratification in patients with pulmonary arterial hypertension (PAH), outperforming single‐parameter assessments.^10^ Similarly, Colak et al. found that, among patients with PAH and right ventricular dysfunction, a decrease in right ventricular outflow tract velocity time integral (RVOT VTI) independently predicts mortality. Each 1 cm reduction in RVOT VTI correlates with a 14.3% increase in the risk of death, further highlighting its prognostic value in this high‐risk population.^11^ Although novel treatments for PAH and Eisenmenger's syndrome have often reduced the need for HLTx, it remains the definitive therapy for selected patients.^12^ In this context, combined HLTx continues to be a life‐saving option for patients with end‐stage cardiopulmonary compromise. However, its incidence remains relatively low, largely due to advances in medical, device‐based and surgical therapies that have refined patient selection. For instance, patients with end‐stage idiopathic PAH often present in a severely compromised state, characterised by pulmonary insufficiency, central cyanosis and right heart dysfunction. Despite the combined involvement of both the pulmonary and cardiac systems, these patients are increasingly managed with BLTx rather than HLTx. This shift is supported by evidence indicating that the right heart and tricuspid valve often remodel towards normal size and function following the normalisation of pulmonary vascular resistance after BLTx.^13^

This analysis aims to present our centre's entire experience with HLTx and to compare outcomes between earlier and more recent Eras.

## Methods

Eighty patients underwent heart‐lung transplantation (HLTx) between January 1983 and December 2010, with follow‐up through June 2024. Two patients were excluded due to missing data.

### Ethical approval

The study conformed to the Declaration of Helsinki and was approved by the Institutional Review Board of Ludwig Maximilian University of Munich (LMU; registration no. 4216).

### Study design

The primary endpoint was mortality. Secondary endpoints included intraoperative time, cold ischemic time, ICU stay, mechanical ventilation duration, infections, transplant indications, donor‐recipient gender mismatch, graft rejection, bronchiolitis obliterans syndrome (BOS), transplant vasculopathy, lymphoproliferative disease and the development of malignancy.

Immunosuppressive regimens consisted of a calcineurin inhibitor (initially ciclosporin, replaced by tacrolimus since 1994), antimetabolites (mycophenolate or azathioprine), mTOR inhibitors (e.g., sirolimus) and glucocorticoids. Drug levels were monitored to guide tapering and steroid‐sparing protocols. For antibody‐mediated or refractory rejection, extracorporeal photopheresis and rituximab were employed.

Cardioplegia solutions included HTK and UW2; lung preservation used Euro‐Collins in earlier years, later replaced by Perfadex. Surgical technique involved median sternotomy, cardiopulmonary bypass, en bloc donor organ explantation, vascular anastomoses and bronchial reconstruction with attention to graft viability and postoperative complications.^14^

### Surgical techniques

HLTx commences with a median sternotomy and establishment of cardiopulmonary bypass (CPB) to maintain circulation and oxygenation. The donor heart and lungs are explanted en bloc, preserving major vessels and airway structures. Implantation entails vascular anastomoses of the donor left atrium to the recipient cuff, pulmonary arteries and ascending aorta, followed by end‐to‐end bronchial reconstruction to ensure adequate blood supply and tension‐free alignment, thereby minimising complications. Myocardial protection utilises cold cardioplegic solutions, while lungs are preserved with specialised solutions. Upon completion of anastomoses and weaning from CPB, haemostasis is secured prior to closure.^15^

Advancements in surgical and preservation techniques have markedly improved outcomes. Lung preservation has evolved from Euro‐Collins to Perfadex solution, which affords superior protection against ischaemia–reperfusion injury. Cardiac preservation has progressed from HTK to UW2 solution, enhancing myocardial viability. Refinements in vascular and airway anastomoses have reduced postoperative complications, including arrhythmias, stenosis and bronchial ischaemia; bronchial artery revascularisation is occasionally employed to enhance graft viability. Improved CPB apparatus has bolstered intraoperative stability and diminished inflammation. Developments in organ transport and preservation have extended permissible cold ischaemic times, facilitating flexible transplant scheduling. Emerging techniques, such as ex vivo lung perfusion, hold promise for graft assessment and reconditioning. Alongside surgical innovations, modern immunosuppressive regimens and perioperative care have contributed to improved long‐term survival following HLTx.^14^

### Statistics

Continuous variables are presented as either median (interquartile range) or mean ± standard deviation, according to their distribution. Categorical variables are expressed as absolute and relative frequencies. The cohort was divided into Era 1 and Era 2 groups and compared using Student's t‐test, Fisher's exact test or chi‐square test, as appropriate. Statistical significance was set at *P* < 0.05. Survival analysis was performed using Kaplan–Meier estimates and compared by log‐rank tests. Hazard ratios with 95% confidence intervals were calculated using Cox proportional hazards regression.

## Results

**Figure 1:**
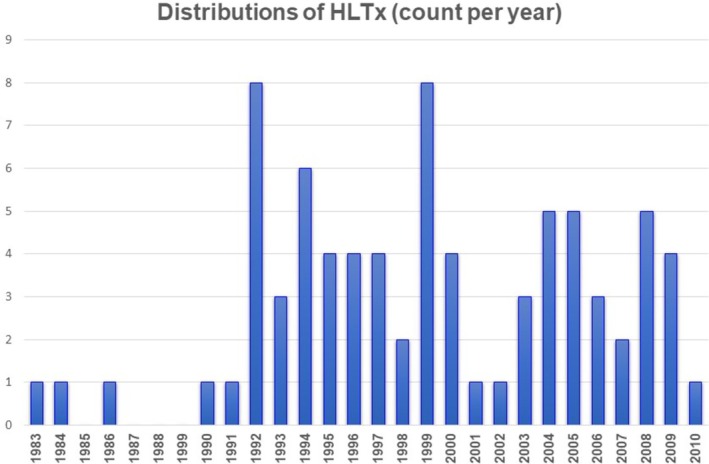
Distribution of HLTx cases per year.

**Figure 2:**
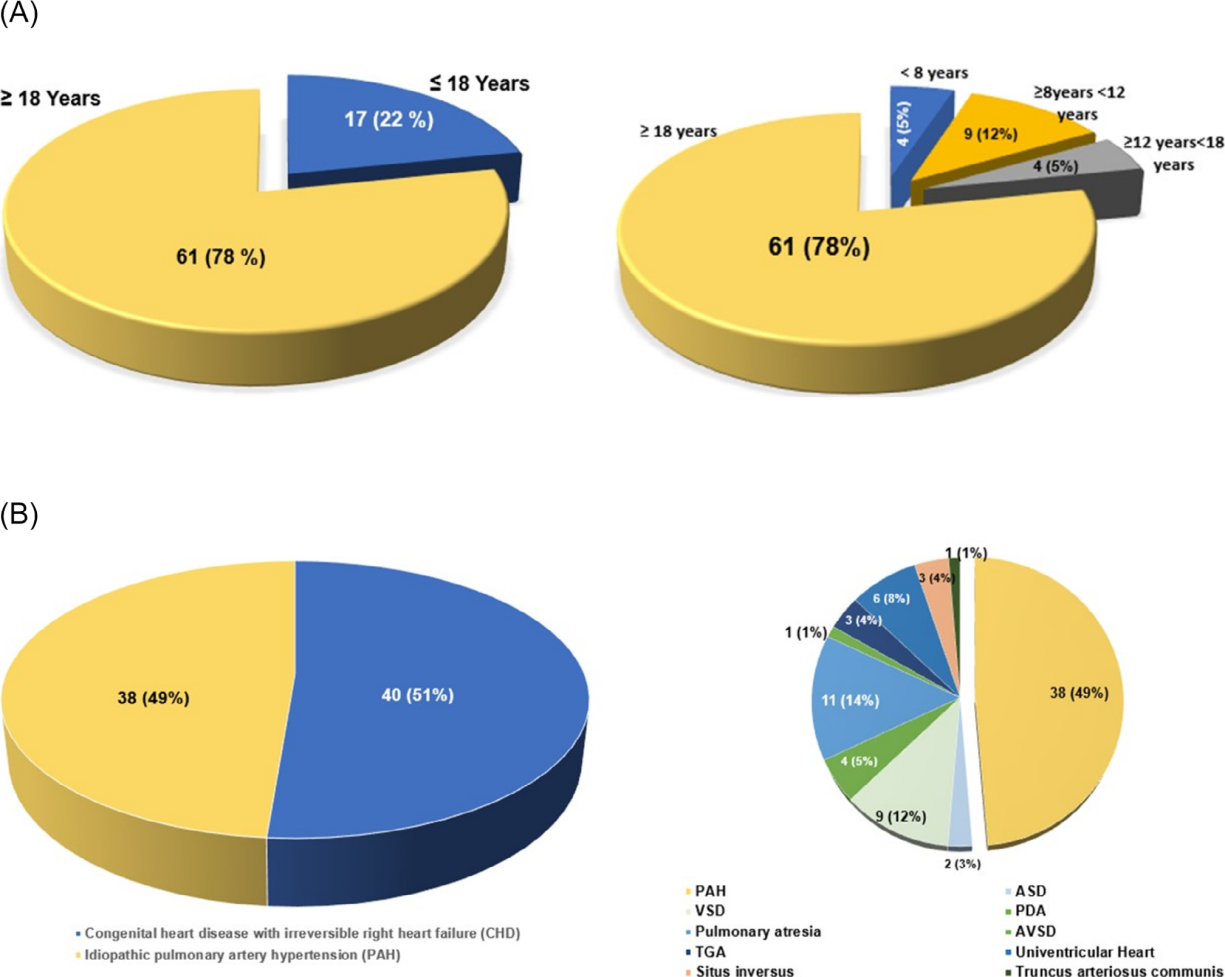
(A) Distribution of recipients' ages. (B) Distribution of diagnosis for transplantation.

Of 80 patients, two were excluded due to missing data. The cohort was divided into Era 1 (1983–1995; *n* = 22, 28%) and Era 2 (1996–2010; *n* = 56, 72%). Baseline recipient and donor characteristics are summarised in *Table* *1*. Follow‐up duration was significantly longer in Era 2 (11 ± 10.5 years) compared to Era 1 (4.5 ± 3 years; *P* = 0.002). Perioperative data and post‐transplant outcomes are detailed in *Table* *2*. Transplant distribution, patient age and diagnosis for transplantation appear in *Figures* *1* and *2* *A,B*. Euro‐Collins was used exclusively for lung preservation in Era 1, while Perfadex predominated in Era 2 (*P* < 0.001). Myocardial protection shifted from HTK in Era 1 to exclusive use of UW2 in Era 2 (*P* < 0.001).

**Table 1:** Recipient's clinical baseline and demographics data at time to HLTx

	Era 1 (1983–1995) = 22 (28%)	Era 2 (1996–2010) = 56 (72%)	*P* value	All (*n* = 78)
	Mean ± SD	Mean ± SD		Median (IQR)
Feature recipient				
Follow‐up (year)	4.5 ± 3	11 ± 10.5	**0.002**	3.71 (0.15–16.64)
Age (year)	30 ± 11	30 ± 15	0.947	30.77 (20.63–41.48)
Gender (male), *n* (%)	9 (41)	28 (50)	0.615	37 (47)
Body weight (kg)	66 ± 14	62 ± 21	0.375	68.00 (59.75–77.25)
BMI (kg/m^2^)	22 ± 3	22 ± 5	0.867	22.62 (19.52–25.77)
Idiopathic PAH	12 (54)	26 (46)	0.617	38 (49)
CHD with irreversible RV failure	10 (45)	30 (54)	0.162	40 (51)
Pre‐operative ECMO/ECLS	5 (23)	11 (20)	1.000	15 (19)
Previous heart operation	4 (18)	13 (23)	0.766	17 (22)
Positive CMV‐IgG	2 (9)	16 (29)	0.079	30 (39)
Positive EBV‐IgG	7 (32)	21 (38)	0.794	23 (30)
Waiting time on list (day)	247 ± 242	251 ± 207	0.569	156.50 (65.0–288.5)
Feature donor				
Age (year)	26 ± 9	28 ± 15	0.458	27.35 (17.66–38.57)
Gender (male), *n* (%)	14 (64)	32 (57)	0.621	46 (59)
Body weight (kg)	68 ± 15	63 ± 21	0.363	70.0 (58.0–79.25)
BMI (kg/m^2^)	24 ± 4	23 ± 5	0.579	23.88 (19.91–26.53)
Feature donor/recipient				
Gender‐mismatch	12 (45)	31 (55)	1.000	43 (55)
BMI‐mismatch	1 (5)	0	0.282	1 (1)
Positive mismatch in CMV‐IgG	5 (23)	22 (39)	0.131	27 (35)
Positive mismatch in EBV‐IgG	7 (32)	22 (38)	0.794	26(33)

Continuous variables are presented as median (IQR) or mean ± standard deviation. Categorical variables are presented as *n* (%). Gender mismatch between donor and recipient is indicated as m/f or f/m. BMI mismatch was defined as a donor‐to‐recipient ratio of <0.8 or >1.2. A mismatch in CMV IgG or EBV IgG status between donor and recipient is indicated as positive/negative or negative/positive, reflecting discordant serostatus.

BMI, body mass index; CHD, congenital heart disease; CMV IgG, cytomegalovirus immunoglobulin G antibodies; EBV IgG, Epstein–Barr virus immunoglobulin G antibodies; ECMO/ECLS, extracorporeal membrane oxygenation/extracorporeal life support; PAH, pulmonary artery hypertension.

**Table 2:** Perioperative data and post‐transplant results

	Era 1 (1983–1995) = 22 (28%)	Era 2 (1996–2010) = 56 (72%)	*P* value	All = *n* (%)
	Mean ± SD	Mean ± SD		Mean ± SD
Cardiac protection			**<0.001**	
HTK	16 (73)	0		16 (21)
UW2	6 (27)	56 (100)		62 (79)
Lung protection			**<0.001**	
Euro‐Collins	22 (100)	4 (7)		26 (33)
Perfadex	0	52 (93)		52 (67)
Ischaemic time (min)	305 ± 94	317 ± 96	0.307	314 ± 95
Post‐Tx ECMO/ECLS	5 (23)	6 (11)	0.276	11 (14)
Length of VS (h)				
≥72 h	11 (50)	30 (54)	0.806	31 (40)
ICU stay (days)	125 ± 66	42 ± 40	0.261	68 ± 46
Bleeding	3 (14)	18 (32)	0.155	21 (28)
Re‐sternotomy	3 (14)	17 (30)	0.158	20 (26)
Kidney dysfunction	7 (32)	19 (40)	1.000	26 (33)
Dialysis	5 (23)	15 (27)	0.781	20 (26)
Hepatic dysfunction	0	10	0.054	10
Acute rejection	14 (64)	27 (48)	1.000	41 (52)
Chronic rejection	5 (23)	6 (11)	0.314	11 (14)
Graft (heart) failure	1 (5)	2 (4)	1.000	3 (4)
CAV	4 (19)	6 (11)	1.000	10 (13)
BOS	8 (36)	21 (38)	**0.045**	29 (37)
PTLD	3 (14)	4 (7)	0.323	7 (9)
Non‐Hodgkin lymphoma	1	0		1
Hodgkin lymphoma	0	1		1
Basal cell carcinoma	0	1		1
Neurological complications	6 (23)	6 (11)	0.108	12 (16)
Bleeding	0	3 (6)	0.098	3 (4)
Stroke	2 (9)	3 (6)	0.768	5 (7)
Diaphragm paresis	1 (5)	7 (13)	**0.040**	8 (10)
Infection				
Legionella	3 (14)	7 (13)	1.000	10 (13)
Bacterial	1 (5)	24 (43)	**<0.001**	25 (32)
Herpes simplex	0	5 (9)	0.314	5 (6)
Candida	3 (14)	17 (30)	0.158	20 (26)
Fugal	0	5 (9)	0.314	5 (6)
Pneumonia	2 (9)	11 (20)	0.330	13 (17)
Aspergillus	2 (9)	14 (25)	0.136	16 (21)
Mycoplasma	4 (18)	11 (20)	1.000	15 (19)
Positive CMV‐IgG	1 (5)	10 (18)	0.166	11 (14)
Positive EBV‐IgG	7 (32)	17 (30)	1.000	24 (31)

Continuous variables: Mean ± standard deviation. Categorical variables: *n* (%).

BOS, bronchiolitis obliterans syndrome (a form of chronic lung allograft dysfunction [CLAD], distinct from restrictive allograft syndrome [RAS]); CAV, coronary artery vasculopathy; CMV‐IgG, cytomegalovirus immunoglobulin G antibodies; EBV‐IgG, Epstein–Barr virus immunoglobulin G antibodies; ECMO/ECLS, extracorporeal membrane oxygenation/extracorporeal life support; HTK, histidine‐tryptophan‐ketoglutarate solution; PTLD, post‐transplant lymphoproliferative disorder; UW2, University of Wisconsin solution; VS, length of mechanical ventilation support (h).

**Figure 3:**
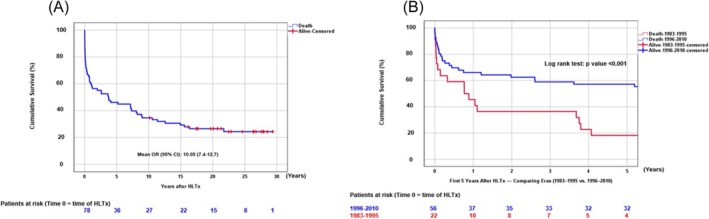
(A) Cumulative survival after HLTx at follow‐up time (years). (B) Cumulative survival after HLTx at 5 years: Comparison of eras (1983–1995 vs. 1996–2010).

**Figure 4:**
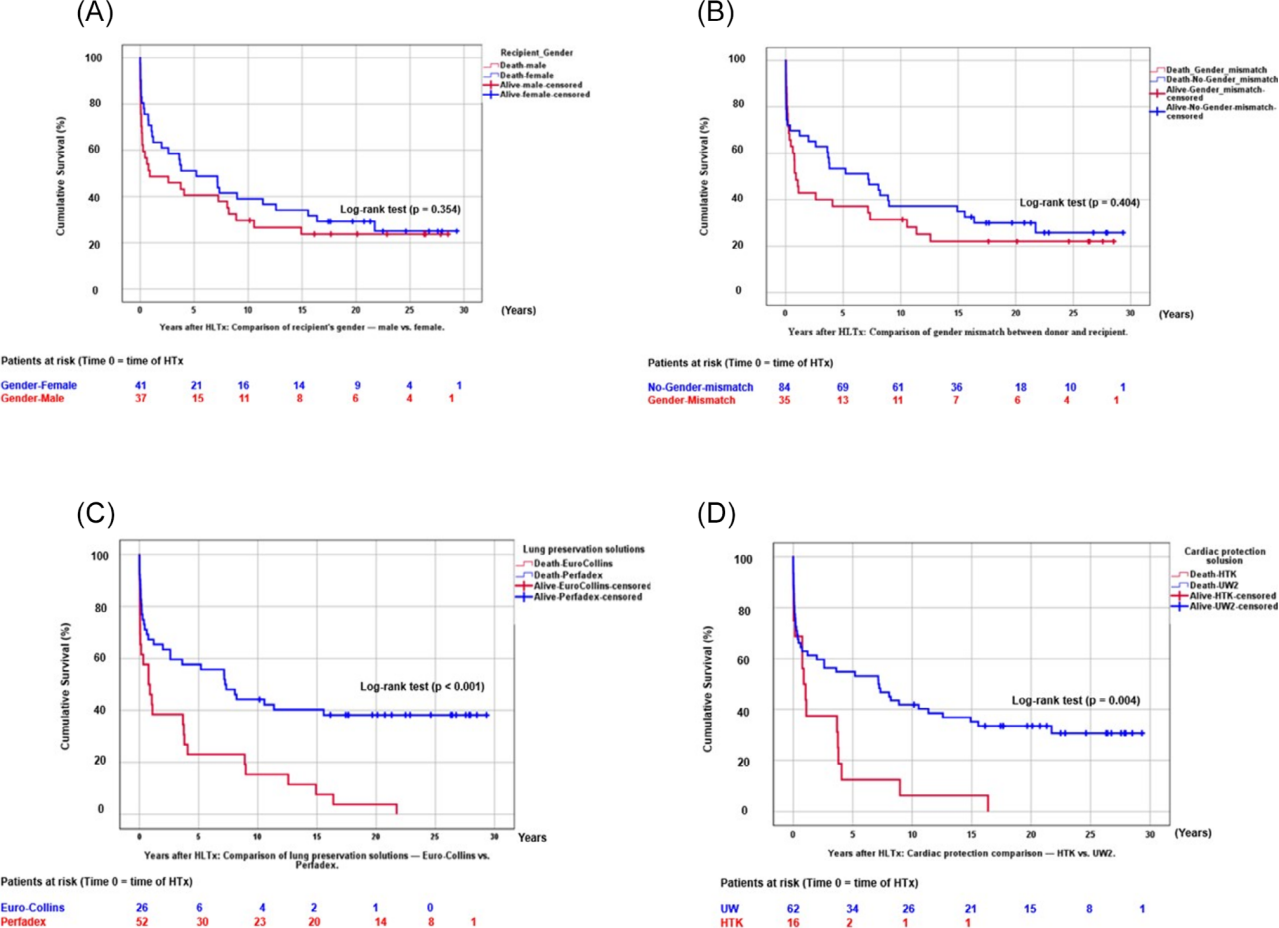
(A) Cumulative survival after HLTx at follow‐up time (years): Comparison of recipient's gender (male vs. female). (B) Cumulative survival after HLTx at follow‐up time (years): Comparison of donor‐recipient gender mismatch (male/female or female/male). (C) Cumulative survival after HLTx at follow‐up time (years): Comparison of lung preservation solution (Euro‐Collins vs. Perfadex). (D) Cumulative survival after HLTx at follow‐up time (years): Comparison of cardiac protection solution (HTK: histidine‐tryptophan‐ketoglutarate).

**Figure 5:**
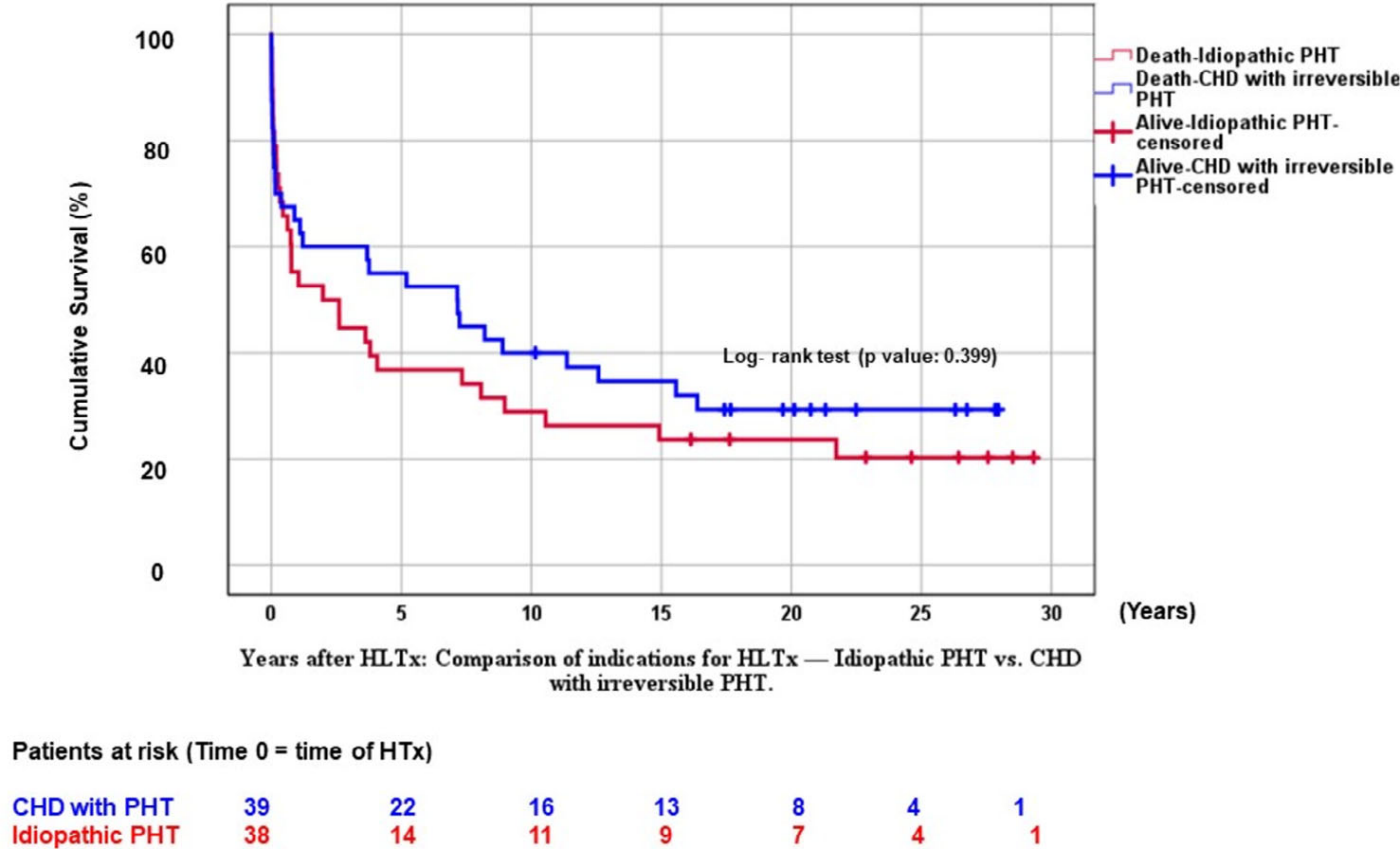
Cumulative survival after HLTx at follow‐up time (years): Comparison of cause of HLTx (idiopathic pulmonary arterial hypertension [PAH] vs. congenital heart disease [CHD] with irreversible right heart failure and secondary PAH).

At last follow‐up, overall survival hazard ratio was 10.05 (95% CI: 7.4–12.7) (*Figure* *3* *A*). Five‐ and 10‐year survival rates were comparable across Eras. There was an increased one‐year survival rate after HLTx in the more recent Era 2 (≈55% and 43%, respectively; *Figure* *3* *B*). Survival did not significantly differ by recipient sex, donor‐recipient gender mismatch or transplant diagnosis (*Figure* *4* *A,B*). Survival improved significantly with Perfadex versus Euro‐Collins and UW2 versus HTK (*P* < 0.001; *Figure* *4* *C,D*). No survival difference was observed between idiopathic PAH and CHD patients (*P* = 0.399; *Figure* *5*). Multiorgan failure and pneumonia (including Aspergillus) each accounted for 15% of deaths. Other causes included single‐organ failure (13%), sepsis (9%), heart failure (9%) and bronchiolitis obliterans syndrome (7%). Intraoperative deaths were 7%; acute pulmonary haemorrhage, circulatory failure and suicide collectively accounted for another 7%.

Patients with ASD, PDA, VSD and AVSD developed Eisenmenger syndrome without prior surgery, while those with TGA, PA, univentricular heart, situs inversus and TAC had failed surgical repairs leading to irreversible PAH. Of 40 CHD patients, 24 had prior surgery, mainly in Era 2. Twenty‐nine CHD and 14 idiopathic PAH patients were referred from other German clinics.

Mean hospital stay was 122 ± 83 days; mean ICU stay ranged from 68 to 46 days. Mean mechanical ventilation lasted 72 h; prolonged ventilation (>72 h) was needed in ~50% of patients in both Eras. Initial immunosuppression commonly combined tacrolimus with mycophenolate mofetil (57%), while other regimens were less frequently used. Nearly half (47.1%) required changes in immunosuppression, evenly split between PAH and CHD groups (*P* = 0.017).

Long‐term complications included coronary artery vasculopathy (13%), chronic cardiac rejection (6%), chronic pulmonary rejection (3%), BOS (37%) and PTLD (9%). PTLD developed in three patients between 4 and 46 months post‐transplant, with three of these patients also diagnosed with non‐Hodgkin lymphoma, basal cell carcinoma and Hodgkin lymphoma. Infections included bacterial pneumonia (17%), CMV pneumonia (14%), Aspergillus (21%), Mycoplasma (19%) and gastrointestinal bleeding (10%). Twenty‐five patients experienced pulmonary rejection episodes.

New BOS cases appeared after month three, peaking at years five and ten, then declining. Previously diagnosed BOS increased from year two, stabilising between years nine and twelve, peaking at ten cases in year ten.

Univariable mortality risk analysis (*Table* *3*) identified transplant Era, use of Perfadex and UW2, and Epstein–Barr virus infection as significant factors. Heart failure: OR 4.557 (95% CI: 1.057–19.648, *P* = 0.042), and gastrointestinal bleeding: OR 2.739 (95% CI: 1.310–5.726, *P* = 0.016). These factors remained significant in multivariable analysis.

**Table 3:** Univariable risk analysis for mortality after HLTx: Cox regression analysis

	Univariable Cox analysis		
	OR (mean)	95% CI	*P* value
Recipient features			
Era	**0.383**	**0.221–0.663**	**<0.001**
Age (per years)	1.009	0.991–1.028	0.316
Gender (male)	0.784	0.468–1.313	0.355
PAH/CHD	0.801	0.478–1.343	0.400
BMI (per kg/m^2^)	1.031	0.969–1.097	0.335
Previous heart operation	0.847	0.439–1.634	0.621
Pre‐HTx ECLS/ECMO	0.960	0.508–1.813	0.899
Waiting on the list (days)	1.001	0.999–1.002	0.309
Positive CMV‐IgG	0.729	0.385–1.379	0.331
Positive EBV‐IgG	0.949	0.548–1.644	0.852
Age (per years)	1.005	0.987–1.024	0.585
Sex (male)	0.996	0.590–1.680	0.988
BMI (per kg/m^2^)	1.032	0.972–1.096	0.303
Positive CMV‐IgG	0.889	0.520–1.519	0.666
Positive EBV‐IgG	0.957	0.515–1.776	0.888
Donor/recipient features			
Mismatch in BMI donor/recipient	0.853	0.118–6.180	0.875
Mismatch in Gender donor/recipient	0.802	0.477–1.348	0.405
Mismatch in CMV‐IgG donor/recipient	0.944	0.549–1.622	0.834
Mismatch in EBV‐IgG donor/recipient	0.668	0.382–1.168	0.157
Post‐transplant features			
RV failure	2.013	0.904–4.482	0.087
Lung failure	1.072	0.428–2.683	0.882
Heart failure	**4.557**	**1.057–19.648**	**0.042**
Lung protection	**0.393**	**0.232–0.666**	**<0.001**
Cardiac protection	**0.425**	**0.234–0.769**	**0.005**
ECMO/ECLS	1.398	0.686–2.848	0.356
Bleeding	0.654	0.331–1.295	0.203
Gastrointestinal bleeding	**2.739**	**1.310–5.726**	**0.016**
Re‐thoracotomy	0.659	0.323–1.343	0.251
Renal dysfunction	1.304	0.756–2.249	0.340
Dialysis	1.524	0.852–2.724	0.155
Infection			
Mycoplasma	1.758	0.940–3.289	0.077
Aspergillus	0.800	0.414–1.545	0.506
Fugal	1.398	0.505–3.876	0.519
Bacterial	0.833	0.473–1.468	0.527
Legionella	1.741	0.822–3.690	0.148
Herpes simplex	2.003	0.790–5.082	0.143
Candida	0.730	0.394–1.354	0.318
Pneumonia	0.013	0.497–2.065	0.971
Neurological complication	1.659	0.832–3.308	0.151
Rejection	1.444	0.857–2.432	0.168
Positive CMV‐IgG	0.630	0.270–1.467	0.284
Positive EBV‐IgG	**1.753**	**1.013–3.032**	**0.045**
CAV	0.715	0.285–1.792	0.474
BOS	0.604	0.093–4.373	0.618
Liver dysfunction	1.050	0.450–2.449	0.910
PTLD	1.542	0.659–3.607	0.318
Ischaemic time per minute	1.000	0.997–1.002	0.886

Univariable and multivariable risk factor analysis for mortality in the cohort was performed using the chi‐square test. BMI mismatch was defined as a donor‐to‐recipient ratio of <0.8 or >1.2. Gender mismatch between donor and recipient is indicated as m/f or f/m. Ischaemic time was defined as the interval from donor heart aortic clamp until reperfusion in the recipient.

BMI, body mass index; BOS, bronchiolitis obliterans syndrome; CAV, coronary artery vasculopathy; CHD, congenital heart disease; CI, confidence interval; CMV‐IgG, cytomegalovirus immunoglobulin G antibodies; EBV‐IgG, Epstein–Barr virus immunoglobulin G antibodies; ECMO/ECLS, extracorporeal membrane oxygenation/extracorporeal life support; HTK, histidine‐tryptophan‐ketoglutarate solution; ICU, intensive care unit; OR, odds ratio; PAH, idiopathic pulmonary arterial hypertension; PTLD, post‐transplant lymphadenopathy disorder; UW2, University of Wisconsin solution.

Multivariable analysis confirmed transplant Era and gastrointestinal bleeding as independent predictors. EBV positivity showed a nonsignificant hazard ratio of 1.542 for PTLD (*P* = 0.318), suggesting no clear association.

## Discussion

This retrospective study analyses outcomes of all HLTx procedures performed at our centre, demonstrating survival rates comparable to international data.^2^, ^5^ Survival in the Era 2 appears improved compared to historical cohorts, although interpretation is constrained by multiple influencing factors. Comparable baseline characteristics suggest that patient selection has remained largely consistent.

In recent times, patients with combined end‐stage pulmonary and cardiocirculatory failure from idiopathic PAH are treated with timely BLTx, which offers comparable or better long‐term outcomes.^6^, ^7^, ^8^, ^9^, ^16^ Normalisation of pulmonary vascular resistance enables right heart and tricuspid valve remodelling.^13^ BLTx is also preferred due to organ scarcity.

Although this study does not isolate the effects of changing immunosuppressive protocols, the implementation of triple immunosuppression with tacrolimus, mycophenolate acid and selective mTOR inhibitors appears to have contributed to improved outcomes.^14^ Multidisciplinary, individualised care remains a critical component of success. Modern organ preservation strategies, including the use of Perfadex for lungs and UW2 solution for cardiac grafts, may have also enhanced outcomes. Meta‐analyses support long‐term survival benefits associated with Perfadex use in organ preservation.^17^ However, sample size and cohort heterogeneity limit definitive conclusions.

Technologies like oxygenated machine perfusion and ex vivo lung perfusion show promising results but have yet to demonstrate efficacy in the context of combined HLTx. Bronchiolitis obliterans syndrome (BOS), a form of chronic lung allograft dysfunction (CLAD), remains a significant long‐term complication, with an incidence of 37%. CLAD also includes restrictive allograft syndrome (RAS). Treatments including extracorporeal photopheresis^18^ and rituximab^19^ show potential for BOS management, though additional evidence is required. BOS incidence was higher in Era 2, likely due to extended follow‐up.^20^ BOS occurs less frequently after HLTx than after isolated lung transplantation. Chronic rejection remains a principal contributor to graft failure. BOS results in progressive airflow limitation and FEV1 decline, despite immunosuppressive treatment.^21^

Chronic immunosuppression, particularly with calcineurin inhibitors, is associated with nephrotoxicity.^22^ In this study, renal dysfunction occurred in 33% of HLTx recipients, with 26% requiring dialysis. In contrast, dialysis rates following isolated LTx ranged from 0.6% at 1 month to 13% at 5 years.^23^ Chronic kidney disease continues to pose a major risk for morbidity and mortality post‐transplant,^24^, ^25^ as reported by Ishani et al.^24^ and Boyle et al.^26^ One patient in our cohort required kidney transplantation 7 years post‐HLTx. Groetzner et al. demonstrated that conversion to sirolimus and mycophenolate improved renal function after lung transplantation.^27^ Neither renal dysfunction nor the need for dialysis was identified as a mortality risk factor in the univariable Cox regression analysis conducted in this study.

Lung transplantation generally yields more favourable outcomes, consistent with registry data and institutional experience. These findings reflect a global decline in HLTx procedures, with isolated organ transplantation now preferred for selected patients.^6^, ^7^, ^13^, ^28^, ^29^, ^30^ HLTx remains appropriate for patients with CHD and irreversible pulmonary complications, such as Eisenmenger's syndrome,^8^ or advanced PAH with right heart involvement unsuitable for isolated lung transplantation.^31^ In our cohort, CHD with irreversible right heart involvement and Eisenmenger's syndrome accounted comparable in both Eras.

Early post‐transplant challenges include multiple forms of graft rejection: hyperacute rejection due to preformed antibodies,^32^ acute cellular rejection, antibody‐mediated rejection resulting in vascular injury,^33^ and ischaemia‐reperfusion injury.^22^ Graft failure and right ventricular failure emerged as mortality risk factors in the univariable Cox regression analysis. Cardiac allograft vasculopathy (CAV), characterised by coronary fibrosis and functional deterioration, is driven by inflammation and endothelial dysfunction.^34^, ^35^ In our cohort, 13% of patients developed CAV, with no significant differences observed between Eras.

Prolonged immunosuppression and high doses increased the risk of PTLD and opportunistic infections.^36^ A rise in bacterial infections was observed in Era 2, underscoring the ongoing challenge of infection prevention. Viral and fungal infections, including CMV and EBV, remained stable. Lifelong immunosuppression reduces immune surveillance, increasing susceptibility to viral complications.^35^ EBV‐IgG seropositivity (*P* = 0.045) was identified as a predictor of mortality in the univariable Cox regression analysis. PTLD itself did not show a significant association with EBV‐IgG seropositivity and mortality in the same analysis.

In the future, palliative interventions such as the reversed Potts shunt may offer clinical benefit for paediatric patients with suprasystemic PAH unresponsive to medical therapy.^36^, ^37^ Atrial flow regulator devices can delay transplantation in paediatric heart failure.^4^, ^14^ When cardiac function is preserved, BLTx is generally the preferred strategy, whereas HLTx is required in cases of irreversible heart failure with severe PAH.^12^ Bridging to transplant remains complex; however, paracorporeal lung assist devices represent a viable alternative to ECMO.^38^, ^39^ Although interventional palliative techniques can reduce right ventricular afterload in paediatric PAH, these procedures carry significant risks and require careful patient selection.^40^, ^41^

### Limitations

Long follow‐up periods and heterogeneity in age, sex and underlying diagnosis limited data consistency, interpretability and generalisability. The retrospective design resulted in missing follow‐up data, primarily due to early post‐transplant mortality or follow‐up conducted at external centres. Data collection time points varied by up to 3 months from the predefined cut‐off dates, introducing additional variability. The small number of paediatric patients introduced age‐related selection bias and reduced the applicability of findings to broader patient populations.

## Conclusions

HLTx outcomes at our centre are consistent with international standards. Improvements in immunosuppressive therapy, organ preservation and perioperative management have contributed to enhanced survival over time. Previously considered a last‐resort intervention, HLTx is now infrequently performed. Patients with CHD and irreversible PAH are managed by specialised multidisciplinary teams, with HLTx reserved for those who have failed multiple surgical therapies. For patients with idiopathic PAH and preserved cardiac function, BLTx remains the preferred treatment strategy.

## Conflict of interest

None declared.

## Funding

None.
